# Characterizing the Bat Virome of Vietnam: A Systematic Review of Viral Diversity and Zoonotic Potential

**DOI:** 10.3390/v17121532

**Published:** 2025-11-22

**Authors:** Vasilina K. Lapshina, Natalia I. Guskova, Ivan F. Stetsenko, Mo T. Luong, Truong V. Tran, Alina D. Matsvay, German A. Shipulin, Sergey M. Yudin, Veronika I. Skvortsova

**Affiliations:** 1 Federal State Budgetary Institution “Centre for Strategic Planning and Management of Biomedical Health Risks” of the Federal Medical Biological Agency, Moscow 119435, Russia; 2 Southern Branch of the Joint Vietnam-Russia Tropical Science and Technology Research Center, Ho Chi Minh City 731000, Vietnam; luongmo@mail.ru (M.T.L.); truongleky@gmail.com (T.V.T.); 3 Federal Medical Biological Agency, Moscow 123182, Russia

**Keywords:** *Chiroptera*, bat virome, viral diversity, zoonotic potential, emerging viruses, viral reservoirs, Vietnam, Southeast Asia

## Abstract

Bats have been identified as reservoir hosts for an exceptional diversity of viruses, including multiple taxa of high zoonotic concern. Over a hundred bat species inhabit Vietnam, which, combined with significant biodiversity, carry high risk of zoonotic spillover due to dense human–animal interfaces, extensive wildlife trade, and proximity to recent outbreak epicenters. This review systematically synthesizes data on the bat virome in Vietnam and neighboring Southeast Asian countries, assessing viral diversity, host species involvement, and zoonotic potential. By prioritizing virus groups with established zoonotic capacity and pandemic potential, the systematic search identified studies reporting viruses from 32 families across 13 bat families. Based on the WHO 2024 risk classification, seven of these viral families were categorized as high-risk, three as medium-risk, and twelve as low-risk. The comparatively higher viral diversity reported in neighboring countries suggests that the current study likely represents an underestimation of the true virome present in Vietnamese bat populations. We emphasize the urgent need for expanded virological studies integrating metagenomic sequencing, serological surveys, and ecological modeling to improve early detection of emerging threats, as the comparatively higher viral diversity reported in neighboring countries suggests existing research likely represents an underestimation of the true virome present in Vietnamese bat populations. Strengthening regional collaboration is critical for establishing proactive pandemic prevention strategies in this high-risk zoonotic hotspot.

## 1. Introduction

Bats are now widely recognized as critical reservoirs for emerging viruses of significant public concern, including zoonotic pathogens such as coronaviruses (SARS-CoV-2, MERS), filoviruses (Marburg virus and Ebola virus), paramyxoviruses (Hendra virus and Nipah virus), rhabdoviruses (rabies virus) [1,2,3]. Metagenomic sequencing has become a pivotal tool for characterizing the diversity of pathogenic microorganisms in bats, enabling the detection of novel and known viruses without prior target selection [4,5,6,7,8]. Given the vast viral diversity in bats and the escalating human-domestic animal-wildlife interface–driven by land-use changes, wildlife trade, and agricultural expansion–it is likely that numerous viruses remain undiscovered, and undocumented spillover events occur frequently [9]. Characterizing the bat virome through systematic surveillance is critical for early detection of emerging zoonotic threats, understanding viral evolution and host–pathogen dynamics, and informing predictive models for pandemic preparedness–particularly in high-risk interfaces where human-bat contact is increasing.

The order *Chiroptera* is the second largest order of mammals, comprising more than 1250 species [10,11] all of which carry a wide variety of viruses. The Indochina biogeographic region is home to more than 150 species of bats [12,13], almost 2/3 of which can be found in Vietnam [11,14], making the bat fauna of Vietnam one of the richest in the paleotropical realm [15]. This exceptional species richness, concentrated in karst landscapes and tropical forests, creates a hotspot for host–virus coevolution and zoonotic emergence risk [11,16,17].

In recent years, there has been a dynamic of data accumulation on the species diversity of bats living in Vietnam. The majority of the recently described species are also found in neighboring countries; the only known exceptions are *Murina harpioloides* [18], *Myotis phanluongi* [19], *Hipposideros griffini* [20] and *Hipposideros alongensis* [21], which were found exclusively in Vietnam. However, it is possible that there are more endemic species that have yet to be described [11].

Bat migration patterns show distinct latitudinal variation. In temperate regions, three behavioral categories are recognized: sedentary species (non-migratory) with limited movements <50 km, regional migrants traveling 100–500 km seasonally, and long-distance migrants covering >1000 km between seasonal ranges [22]. While tropical bats generally exhibit more restricted movements, exceptions exist–notably *Chaerephon plicata* populations in Vietnam that undergo 300 km seasonal migrations between Guangxi (China) and Hainan Island [23]. Given the limited long-distance dispersal typical of tropical bat species [24], our investigation of Vietnam’s bat virome focused on studies from ecologically contiguous regions: Vietnam, Laos, Cambodia, and southeastern China. This geographic scope reflects shared chiropteran fauna while minimizing inclusion of evolutionarily distinct populations.

While several isolated studies in Vietnam have reported fragmented data on specific viral family, such as *Coronaviridae* [25,26,27,28] or *Hantaviridae* [29,30,31,32], a comprehensive and systematic characterization of the total bat virome remains absent. This lack of a unified overview hinders a holistic understanding of viral diversity, co-circulation patterns, and the full spectrum of zoonotic risk, creating a critical gap this study aims to fill. The article explores the potential virome diversity of bats in Vietnam, summarizing both the data obtained in the territory of Vietnam and in the bordering areas included in the habitat of bats of the fauna of Vietnam. Our findings underline bats’ critical role as reservoirs for numerous viruses, including high-priority pathogens, that cause severe diseases in humans and have high pandemic potential, such as *Coronaviridae*, *Filoviridae*, *Paramyxoviridae* [33]. This reservoir host characterization provides critical data for regional biosurveillance programs, predictive modeling of spillover risks, and targeted prevention of zoonotic emergence in Southeast Asia’s pandemic hotspots.

## 2. Materials and Methods

The systematic literature search was conducted in accordance with the Preferred Reporting Items for Systematic reviews and Meta-Analyses (PRISMA) guidelines [34]. The review was conducted in four stages: database search, assessment of relevant articles, data extraction and summarization.

### 2.1. Eligibility Criteria

Eligibility criteria were established prior to the screening process. This review incorporated studies utilized virological (virus isolation/culture), serological (antibody detection), molecular (PCR-based screening), and metagenomic (sequencing) approaches to assess viral infections in bats. To account for potential bat dispersal and ecological connectivity, the geographical scope was expanded beyond Vietnam to include contiguous regions where Vietnamese bat populations are known to range. These included areas of Laos with shared karst ecosystems, Cambodia’s Mekong floodplain corridors, and the adjacent provinces of Guangxi and Yunnan in southern China.

The review included original research articles, published in English between 2002 and 27 June 2025, that reported viral detection in bats using the specified methodologies. Studies were eligible for inclusion if they were conducted within Vietnam or the previously defined adjacent regions and provided data pertinent to the Vietnamese bat fauna, including bat taxonomy, sampling locations, and identified viral families. Information on bat biodiversity in Vietnam was primarily sourced from the works of Kruskop [11,14].

Studies were excluded if they were review articles, phylogenetic or morphological analyses, or if they lacked original and complete viral detection data. Research focusing on ecologically isolated bat populations, such as those on Malaysian islands, was omitted due to the pronounced zoogeographic divergence between the Sundaic (island) and Indochinese (mainland) faunal regions [11,35,36,37]. Studies from Thailand were also excluded, as the bat fauna exhibits minimal overlap with Vietnam, with only Craseonycteris thonglongyai (the bumblebee bat) being a shared species [11]. Finally, research from Myanmar was excluded based on the absence of regular transboundary bat movements between Myanmar and Vietnam, a dispersal barrier attributed to the extensive agricultural landscapes of intervening Laos [38].

### 2.2. Sources of Information and Search Strategy

The PubMed database served as the primary source for identifying relevant studies published over the past 23 years. The search strategy incorporated key terms including the words “bat(s)”, “Chiroptera”, “chiropterans”, “virus(es)”, “zoonotic pathogens”, “zoonotic diseases”, “Vietnam”, “Viet Nam”, “Southeast Asia”, “Asia”. The final search was executed on 27 June 2025. To ensure comprehensive coverage, the reference lists of all included articles were manually screened.

### 2.3. Article Choice and Data Collection

Study selection followed a two-stage screening process: (1) initial title followed by (2) a full-text assessment. Two reviewers conducted these steps independently; any discrepancies were resolved through consensus or by consultation with a third reviewer. The Covidence system [39] was used to manage this process.

Data extraction was performed manually using a standardized form to collect publication details, sample characteristics, and identified virus families or species. If species identification was not available, analysis was performed at the genus or family.

### 2.4. Data Items

All studies reporting virus detection in bats within the country or ecologically contiguous regions were included in the final analysis. The following data were extracted from each study: publication year, sampling year and country, total and infected bat samples, virus family, bat family, and DOI (Supplementary Materials, Table S1). Viral families detected in Vietnamese bats were summarized and descriptively classified according to the priority pathogen list. Results were mapped to visualize the distribution of viral families. We aimed to match viruses with their potential bat hosts (Table S2); however, incomplete reporting and methodological variability limited this comparison.

### 2.5. Study Risk of Bias Assessment

A formal risk of bias assessment was not performed because the aim of the review was to qualitatively describe virus detections rather than to estimate prevalence or statistical associations. The potential for underestimation of viral diversity resulting from these methodological heterogeneities is acknowledged as an inherent limitation and is addressed in the Discussion.

### 2.6. Effect Measures and Synthesis of Results

Quantitative effect measures were not calculated, as the analysis was descriptive in nature. A qualitative synthesis of the data was performed, with results presented in tabular form and the distribution of viral families visualized on a regional map. Meta-analytic methods were not applied due to the heterogeneity of study designs among the included research.

### 2.7. Reporting Bias and Certainty Assessment

The possibility of reporting bias remains, as the analysis was limited to English-language articles indexed in PubMed and did not include unpublished or local data sources.

The certainty of the evidence is considered moderate to limited, due to heterogeneity in methodologies, variations in diagnostic test sensitivity, and the lack of standardized protocols for bat sampling and virus testing.

## 3. Results

The initial screening process identified 726 unique publications for inclusion in the review. Following title and abstract screening, 361 studies were retained for full-text assessment. Of these, 127 full-text articles were evaluated for eligibility. A total of 67 studies were excluded. Inappropriate study design (*n* = 49)–including articles that analyzed previously published viral sequences or provided theoretical or modeling frameworks rather than original detection data [40,41,42,43,44]. Collection outside the study region (*n* = 12)–studies conducted beyond the defined geographical scope [45,46]. Non-native species (*n* = 6)–studies investigating bat species not known to inhabit Vietnam [47,48].

Following a full-text assessment, 60 publications met inclusion criteria and were retained for in-depth analysis. All included publications represented original research articles that reported viral detections in bats. All selected studies were thoroughly reviewed and systematized; detailed extracted data are provided in the Supplementary Materials (Table S1). A PRISMA flow diagram illustrating the process of study identification, screening, and inclusion is presented in the Supplementary Materials (Figure S1).

A formal assessment of risk of bias in studies, reporting biases, and certainty of evidence was not performed due to the high heterogeneity of the included studies. The main potential sources of bias were related to uneven geographical and taxonomic coverage, differences in diagnostic sensitivity, and incomplete reporting of negative results.

### 3.1. Bat Virome Composition Overview

In our assessment of the bat virome in Vietnam, we prioritized virus families with known public health significance. A total of 32 virus families were identified, including unclassified taxa, of which 25 families are known to be able to infect mammals, with the remaining 7 being either related to the bat diet (insects or plants) or bat microbiome.

The studied bat population comprised predominantly insectivorous species (~70%). While virome studies have revealed diet-associated and microbiome-linked viruses [6,7], our analysis specifically focused on mammalian-infecting viruses due to their zoonotic potential. According to the WHO (2024) priority pathogen classification [33], the viral families were categorized into four risk tiers based on their epidemiological threat level and zoonotic capacity related to Public Health Emergencies of International Concern (PHEICs), providing a basis for targeted surveillance of high-risk pathogens (Table 1). Other virus families are discussed in the section “Virus Families with No Demonstrated Pandemic Potential”, chapter 3.5.3. “Other taxonomic groups”.

Of the mammalian-infecting viruses detected, 11 belonged to DNA virus families while 21 (including unclassified taxa) were RNA viruses. This apparent predominance of RNA viruses likely reflects methodological biases in sample processing rather than true biological prevalence, as bats are known to harbor exceptionally diverse viral communities encompassing all genomic architectures [2,97]. Among the identified viruses, four families pose particularly high zoonotic risks: *Coronaviridae*, *Filoviridae*, *Paramyxoviridae*, and *Rhabdoviridae*. These families contain well-documented pathogens capable of cross-species transmission from bats to humans, often causing severe disease with high case fatality rates. Representative viruses include SARS-CoV-2 (*Coronaviridae*), Ebola virus (*Filoviridae*), Nipah virus (*Paramyxoviridae*), and rabies virus (*Rhabdoviridae*), all of which have caused significant human outbreaks with high mortality rates [1,2,3,33,98]. Post-COVID-19 research focus is evident in the disproportionate attention given to *Coronaviridae* (21/60 studies). Our findings not only map Vietnam’s bat virome but reveal critical knowledge gaps, particularly regarding understudied but high-risk families. Prioritizing longitudinal surveillance and viral characterization will be crucial for pandemic preparedness.

**Figure 1:**
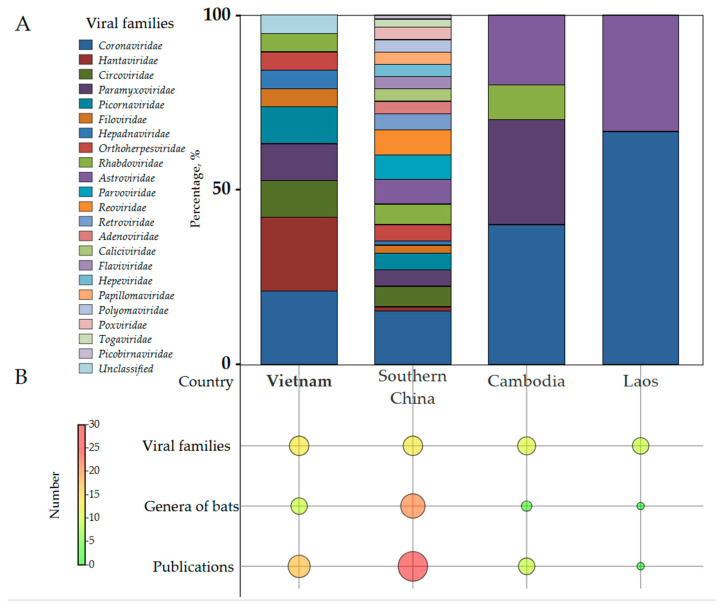
Comparative analysis of bat virome across Southeast Asia. (**A**) The top panel shows normalized composition of the bat virome in Vietnam, Southern China (Guangxi and Yunnan provinces), Laos, and Cambodia. (**B**) The bottom panel presents bibliometric and diversity metrics, showing the number of publications and the corresponding richness of identified bat virus families and genera for each region.

Our analysis of Vietnam’s bat virome incorporated viral data from neighboring regions (Laos, Cambodia, and southeastern China) (Figure 1). This approach was justified by the limited dispersal capacity of tropical bat species [24], which constrains their geographical range and makes adjacent ecosystems biologically relevant for assessing regional viral diversity. By including these proximate areas, we provide a more comprehensive evaluation of potential zoonotic threats. The complete dataset used for visualization is presented in Supplementary Table S2.

The figure demonstrates the number of publications documenting viral diversity of the corresponding countries as well as the numbers of bat genera and viral families found in these countries. Vietnam exhibits notably high bat genus diversity (14 genera), ranking among the highest in Southeast Asia. Nevertheless, both the volume of research and the number of characterized viral families in Vietnam remain substantially lower than in China, suggesting the need for additional research in Vietnam. Furthermore, our analysis identifies a pronounced research bias towards the *Coronaviridae* family across all studied regions, a trend attributable to intensified global surveillance following the COVID-19 pandemic. Consequently, coronaviruses were not only the most frequently detected viral family but also the only one identified in every geographic area included in this review.

Notably, China and Vietnam are the only countries in the region where highly pathogenic viruses from the families *Filoviridae* and *Hantaviridae* have been identified. Our analysis revealed a substantial disparity in overall viral diversity: China exhibited the greatest richness with 22 out of the 23 total families, while Vietnam demonstrated markedly lower diversity (11 families). The limited diversity detected in Laos and Cambodia (2 and 4 families, respectively) likely reflects a combination of insufficient surveillance effort and genuine ecological differences in their bat viromes.

Vietnam shares a number of common viral families with China and other neighboring countries, beyond the previously mentioned *Coronaviridae*. Specifically, seven overlapping families have been identified with China (*Coronaviridae*, *Hantaviridae*, *Circoviridae*, *Picornaviridae*, *Filoviridae*, *Hepadnaviridae*, *Orthoherpesviridae*), and three with Cambodia (*Coronaviridae*, *Paramyxoviridae*, *Rhabdoviridae*), highlighting the interconnectedness of regional bat populations and the potential for cross-border virus circulation.

Eleven viral families reported in more than one study in southern China were absent in Vietnam and other neighboring countries, these families, including *Adenoviridae*, *Caliciviridae*, *Flaviviridae*, *Hepeviridae*, *Papillomaviridae*, *Parvoviridae*, *Polyomaviridae*, *Poxviridae*, *Spinareoviridae* and *Sedoreoviridae* (previous: *Reoviridae*), *Retroviridae*, and *Togaviridae*. Notably, and in contrast to all adjacent countries, representatives of the *Astroviridae* family have not yet been detected in Vietnam, suggesting a high likelihood of its future discovery. Considering that tropical bat species possess considerable migratory ranges capable of bridging national borders, the apparent absence of certain viral taxa in Vietnam likely reflects sampling and characterization gaps rather than true ecological absence. This underscores the need for systematic, family-agnostic virome studies to properly evaluate the spectrum of zoonotic threats in this biodiverse region.

**Figure 2:**
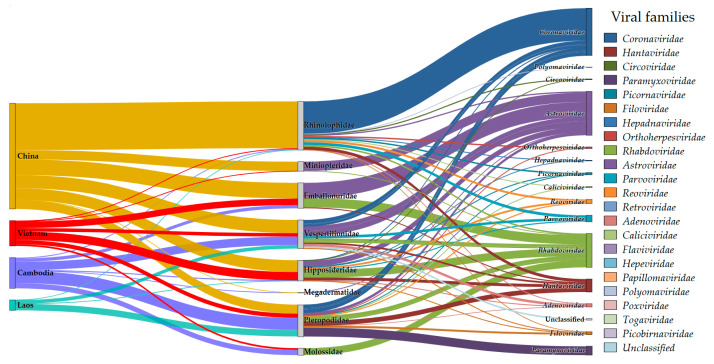
Sankey diagram of bat-associated virus transmission networks. The visualization depicts flow relationships between sampling countries (**left nodes**); bat host families (**center nodes**); detected virus families (**right nodes**). Viral families are color-codded (see legend). Node heights correspond to relative host/virus diversity. Flow bandwidth reflects a number of confirming studies.

To evaluate bat species-associated viral diversity, we conducted a systematic analysis of host–virus relationships across 40 selected studies meeting our inclusion criteria. These studies provided: host taxonomy (species/genus-level identification), infection prevalence data (number of infected individuals). No effect estimates were calculated, as data were binary (virus detected/not detected). The analysis applied these exclusion criteria: pooled-sample studies (precluding individual prevalence calculation) [83], host identification above genus level (suborder/family only) [27,68,81], environmental detection without host association [26,50,68]. Geospatial analysis of the compiled data identified virus-infected bat populations whose ranges likely include Vietnam (Figure 2). The complete dataset underlying this analysis, including host–virus associations and geographic coordinates, is provided in Supplementary Table S3.

The analysis reveals distinct patterns of viral family diversity across bat taxa in the studied region. *Rhinolophidae* bats harbored the greatest viral diversity (14 families), followed by *Hipposideridae* (12), *Pteropodidae* (11), and *Vespertilionidae* (8)–a pattern that may reflect both true ecological differences and sampling intensity. The diagram suggests the importance of monitoring *Paramyxoviridae* in *Pteropodidae*. Our analysis reveals that all identified *Paramyxoviridae* viruses in Vietnam, Southern China, and Cambodia, with confirmed host associations, are exclusively linked to bats of the *Pteropodidae* family. This specific tropism persists despite regional surveillance having also screened other chiropteran families for these viruses [7,64,65]. Conversely, several studies not included in this analysis (e.g., [8,26]) have reported *Paramyxoviridae* detection in guano pools from other bat families, such as *Rhinolophus* and *Scotophilus*. These findings, which lacked explicit sample-to-virus links, suggest that systematic studies could reveal a broader host range for these viruses than currently established. In addition, the diagram illustrates the broad distribution of *Rhabdoviridae* and *Hantaviridae* across bat populations in the region.

The study specifically highlights several viral families of significant public health concern. Particular research focus has been directed toward the *Coronaviridae* family following the COVID-19 pandemic, due to association with *Rhinolophidae* bats. As well as the *Rhabdoviridae* family, which includes the rabies virus, causes a severe and fatal disease in mammals, including humans. This virus–host relationship has become a priority area in zoonotic disease surveillance. The *Astroviridae* family warrants particular attention, as it remains undetected in Vietnam despite being reported in all adjacent countries. Its known presence in bat genera native to Vietnam strongly suggests its eventual discovery there, highlighting a critical target for future surveillance.

Vietnam demonstrates a distinct bat diversity profile compared to neighboring countries, with *Hipposideridae* and *Emballonuridae* emerging as the predominant genera. These taxa serve as key reservoirs for two viral families of particular concern: *Astroviridae* and *Rhabdoviridae*. These findings collectively highlight the urgent need for comprehensive, region-wide bat virome surveillance in Vietnam.

### 3.2. High-Priority Viral Families with Pandemic Potential

Based on WHO priority pathogen criteria [33], bat-associated virus families mentioned below contain members posing significant international public health risks.

#### 3.2.1. *Coronaviridae*

*Coronaviridae* family comprises three evolutionarily distinct subfamilies with specific host ranges: *Orthocoronavirinae* (affect mammals and birds), *Letovirinae* (restricted to amphibians), and *Pitovirinae* (exclusive to aquatic species) [99,100,101]. Within *Orthocoronavirinae*, the subfamily of greatest public health concern, bats serve as primary reservoirs for two critical genera: *Alphacoronavirus* and *Betacoronavirus*. Betacoronaviruses include several high-consequence zoonotic pathogens–SARS-CoV, MERS-CoV, and SARS-CoV-2 (the causative agent of COVID-19)–all recognized as priority threats under the WHO’s Pandemic Preparedness Initiative [33].

Bats serve as the primary natural reservoirs for Betacoronaviruses, including the likely progenitors of SARS-CoV-2, which originated from horseshoe bats (*Rhinolophus* spp.) [62], including the species *R. affinis* and *R. malayanus*, which are distributed not only in China (Yunnan), but also in Vietnam, Laos, Myanmar [11]. This broad distribution indicates that Southeast Asia, particularly Vietnam, may represent a key zone for the evolutionary diversification and potential spillover of SARS-related coronaviruses. Similarly, SARS-CoV is associated with Chinese horseshoe bats (*Rh. sinicus*) [56]. Researchers have isolated live SARS-like coronaviruses (95% identical to human strains) from *Rh. sinicus* that can directly infect human cells via ACE2 receptors, confirming this species as the evolutionary origin of SARS-CoV.

Comparative genomic analyses revealed that alphacoronaviruses exhibit significantly greater genetic diversity than betacoronaviruses in bat reservoirs. This pattern is particularly evident in Vietnamese bat populations, where host-specific coronavirus associations have been documented. Thus, *Miniopterus* and *Myotis* bat genera are infected exclusively with alphacoronaviruses, while bats from the genera *Pipistrellus*, *Tylonycteris* and *Rhinolophus* are carriers of both types of coronaviruses [53]. Such diversity underscores the ecological complexity of coronavirus maintenance in natural reservoirs and indicates potential hotspots for viral recombination and host-switching events.

In Cambodia and Laos, significant diversity of bat coronaviruses was found, including species found in Vietnam, which creates conditions for cross-border circulation of viruses [54]. Surveillance studies in Cambodia and Laos have revealed substantial diversity of bat coronaviruses, including several viral species that overlap with those identified in Vietnam. This epidemiological pattern creates favorable conditions for cross-border viral circulation and suggests their presence in Vietnam [61]. In China, coinfection with different alphacoronaviruses has also been detected in *Rhinolophus* [61], highlighting the risk of viral recombination.

The 2013–2014 surveillance conducted in Vietnam as a part of Emerging Pandemic Threats PREDICT project revealed a high prevalence of coronaviruses in guano farm bats (58 out of 70 sampling sites), especially in the suborder *Microchiroptera* [27]. The overall proportion of positive results (74.8%) was higher than it was in previous studies in Vietnam and neighboring countries [26,54,102]. The 2013–2017 One Health cross-sectoral surveillance in Vietnam expanded the results of PREDICT project and revealed the circulation of viruses with high zoonotic potential in bats, pigs, and humans [25,28]. Significant viral diversity, including alpha- and betacoronaviruses, has been found in bats roosting in areas of human–animal contact, suggesting a high risk of coronavirus transmission from bats to pigs, especially given the popularity of eating both bats and pigs.

Orthocoronaviruses are transmitted through aerosols, the feco-oral route, and contact with contaminated surfaces, causing a spectrum of symptoms from asymptomatic to fatal. The combination of high bat diversity, bushmeat consumption practices, and frequent human–animal contact creates ideal conditions for coronavirus transmission and evolution, necessitating systematic virological surveillance and epidemiological monitoring. These factors are particularly concerning given their demonstrated role in previous coronavirus outbreaks, including SARS-CoV-1 and SARS-CoV-2. Targeted study of these interfaces aligns directly with global pandemic preparedness objectives by identifying high-risk viral strains before they emerge in human populations, characterizing transmission pathways at critical human–wildlife interfaces, and informing early warning systems for potential spillover events [33]. Such proactive measures are essential for preventing future pandemics.

#### 3.2.2. *Paramyxoviridae*

The *Paramyxoviridae* family, as classified by the International Committee on Taxonomy of Viruses, comprises 78 species organized into four subfamilies; some representatives are pathogenic for humans and animals. For humans, the viruses of the *Orthoparamyxovirinae* subfamily are dangerous: measles virus (genus: *Morbillivirus*), Nipah and Hendra viruses (genus: *Henipavirus*) and *Rubulavirinae*: mumps and parainfluenza (genus: *Orthorubulavirus*) [103]. Transmission of the virus is primarily airborne [104].

In recent years, paramyxoviruses have caused a number of outbreaks of zoonotic infections in humans with fatal outcomes–Hendra and Nipah viruses [105,106]. These pathogens have caused recurrent epidemics across Asia, including Malaysia, India, Bangladesh, and the Philippines [107]. The primary natural reservoir for Nipah virus is fruit bats of the genus *Pteropus* (flying foxes). While *Pteropus* species distribution in Vietnam remains sporadic [11], their presence maintains a persistent outbreak risk given the viruses high fatality rates (40–75% for Nipah, 50–75% for Hendra).

Virological surveys have identified *Paramyxoviridae* infections in 15 bat species native to Vietnam, with Nipah virus specifically detected in 12 species across Vietnam and Cambodia [64,65,68]. A Chinese study revealed infections in five ecologically diverse species: insectivorous bats (*Hipposideros cineraceus*, *Hipposideros armiger*, *Taphozous melanopogon*) and fruit bats (*Eonycteris spelaea*, *Rousettus leschenaulti*) [66].

Bats infected with paramyxoviruses typically exhibit minimal clinical symptoms while maintaining efficient viral transmission capabilities to susceptible hosts through multiple routes. Asymptomatic maintenance coupled with high transmission potential–makes bats particularly effective reservoir hosts. The World Health Organization consistently recognizes this threat, maintaining Nipah virus on its annual Research and Development Blueprint list of priority pathogens [33,108].

#### 3.2.3. *Hantaviridae*

The *Hantaviridae* family includes eight genera, members of the *Orthohantavirus* genus can infect humans, causing mild to severe, sometimes fatal illnesses [109]. Humans become infected by inhaling aerosols or secretions from infected animals, or by direct contact. The two hantaviral diseases recognized by WHO are hemorrhagic fever with renal syndrome and hantavirus cardiopulmonary syndrome, which is often fatal [110,111]. The first disease is caused by Dobrava, Hantaan, Puumala and Seoul viruses; the second disease is caused by viruses from North and South America, such as Andes and Sin Nombre viruses [112].

Bat-associated hantaviruses are classified into the genera *Loanvirus* and *Mobatvirus*. Vietnam has emerged as an important research site for bat-borne hantaviruses, with multiple studies conducted [29,30,31,32]. The 2013 virome investigation of 12 bat species across Vietnam and Mongolia led to the discovery of Xuan Son virus (XSV), a novel member of the *Mobatvirus* genus, in *Hipposideros pomona* from Xuan Son Nature Reserve [29]. Subsequent analysis revealed XSV detection in six out of 44 archival *Hipposideros pomona* samples [30] and three of six samples of *Hipposideros cineraceus* bats captured in Vietnam [32].

PCR analysis of archival bat samples from southern and southwestern China revealed the presence of two bat-borne hantaviruses: Laibin virus and XSV [69]. The infection was present in *Taphozous melanopogon*–2.6% (1 of 39) and in *Hipposideros pomona* from Guangxi–9.1% (5 of 55) and from Yunnan–7.5% (3 of 40).

In addition to XSV, a new hantavirus, named Dakrong virus, was found in bats in Vietnam. In an extensive search for hantaviruses in samples isolated from the lung tissues of 215 representatives of the order *Chiroptera*, collected in Vietnam between 2012 and 2014 [31]; a new hantavirus was found in samples obtained from *Aselliscus stoliczkanus* [31].

These findings demonstrate the widespread co-circulation of phylogenetically diverse bat hantaviruses across northern Vietnam [29,30,32] and southern China, delineating a distinct biogeographic zone of viral maintenance spanning the Vietnam-China border region. Of particular concern is the close genetic and antigenic relationship between these bat-borne viruses and known human-pathogenic Hantaviruses. This highlights the need for extensive research to identify the potential emerging hantavirus-related public health threats.

#### 3.2.4. *Filoviridae*

Viruses of the *Filoviridae* family comprise viruses that infect fish, mammals, and reptiles, classified into nine genera, including *Orthoebolavirus* (Ebola virus) and *Orthomarburgvirus* (Marburg virus) [113]. These pathogens cause severe hemorrhagic fevers in humans with a mortality rate of up to 90% [114,115] and are included in the list of priority pathogens by the WHO [33]. Human transmission typically occurs through contact with infected animals or exposure to body fluids of symptomatic individuals [116]. Growing evidence suggests that bats serve as natural reservoir hosts for filoviruses [2,97].

In Vietnam, the key carriers of filoviruses are bats of the genera *Rousettus*, *Eonycteris* and *Cynopterus* [71]. Molecular studies have identified filovirus RNA in *Rousettus leschenaultii* and *R. amplexicaudatus* in a province in northern Vietnam. Phylogenetic analysis revealed two distinct groups: the genus *Dianlovirus* and a clade closely related to *Orthomarburgvirus*. Serological screening detected antibodies against these viruses in 9% of *Rousettus*, 13% of *Eonycteris*, and 10% of *Cynopterus* bats [71].

The findings from bat populations in Vietnam align with studies conducted in bordering regions of China [67,70]. Among genetically distinct filoviruses identified in Chinese bats, a novel virus from the genus *Dianlovirus* was isolated in *Rousettus* species [67]. While no human clinical cases of dianlovirus infection have been reported, the detection of specific antibodies in bat hunters from India and China provides serological evidence supporting potential zoonotic transmission [117]. Another Chinese study detected ebolavirus antibodies in *R. leschenaulti*, *Pipistrellus pipistrellus*, and *Myotis* sp. bats [70]. Key filovirus reservoirs in the region include *Myotis horsfieldii* (distributed in Vietnam) and M. *schreibersii* (taxonomically revised: *M. fuliginosus* has been identified as a separate species, though its distribution in Vietnam requires further verification) [11]. These species, along with *R. leschenaultii* and *Eonycteris spelaea*, represent important filovirus hosts.

#### 3.2.5. *Flaviviridae*

The *Flavivirus* family includes 4 genera: *Orthoflavivirus*, *Pestivirus*, *Hepacivirus*, *Pegivirus*, with different biological properties [118]. Among these, *Orthoflavivirus* genus contains several clinically significant human pathogens, including *Orthoflavivirus zikaense*, *Orthoflavivirus dengue*, *Orthoflavivirus flavi*, *Orthoflavivirus encephalitidis* and *Orthoflavivirus nilense* [33]. These neurotropic viruses frequently cause severe disease manifestations, particularly central nervous system involvement.

Numerous flaviviruses have been isolated from bats [97,119], though their intermediate host-vector relationships and pathogenic potential remain largely uncharacterized. Metagenomic analyses of bat samples collected from southern and southeastern China identified *Flaviviridae* viral sequences in *Rhinolophus affinis* and *Rhinolophus sinicus* [5,6,8]–bat species that are also present in Vietnam [11]. Another study identified *Flaviviridae* viral sequences in 22% of bat samples, demonstrating significant prevalence among these reservoir hosts [6].

It has been established that bats are one of the major reservoirs of pegiviruses and hepaciviruses [45]. Representatives of the genus *Pegivirus* are associated with persistent infections in a wide range of mammalian species, but a connection with disease has not been identified [118]. In contrast, the *Hepacivirus* genus includes clinically significant pathogens, most notably hepatitis C virus (HCV) which causes progressive liver disease in humans [118]. Metagenomic analyses identified a bat *Pestivirus* in *Microchiroptera* [5,8]. Phylogenetic characterization suggests this bat-derived virus may share evolutionary ancestry with known livestock pathogens [8]. Another study represents the first successful isolation of Japanese encephalitis virus (JEV) from *Rhinolophus sinicus*, expanding the known host range for this medically important arbovirus [6]. Despite the high prevalence of *Flaviviridae* in bats, key aspects of their biological and epidemiological significance remain unclear, as demonstrated by the ongoing identification of new susceptible bat species.

#### 3.2.6. *Poxviridae*

The *Poxviridae* family includes two subfamilies: *Chordopoxvirinae* (vertebrate-infecting poxviruses) and *Entomopoxvirinae* (insect-specific poxviruses). Members of the *Chordopoxvirinae* subfamily include several medically important pathogens (*Molluscipoxvirus*, *Orthopoxvirus*, *Parapoxvirus*, and *Yatapoxvirus* genera) capable of causing human disease, typically characterized by cutaneous manifestations ranging from localized pustular lesions to disseminated vesiculopapular rashes. In severe cases, systemic infection can lead to life-threatening complications [120]. In 2022, the World Health Organization (WHO) declared an ongoing mpox disease outbreak [121,122,123]. Mpox is a zoonotic disease caused by *Orthopoxvirus monkeypox* (*Monkeypox virus*), a member of the Orthopoxvirus genus that exhibits structural and antigenic similarities to *Variola virus* (the causative agent of smallpox). Historically, smallpox was a highly contagious disease with mortality rates reaching 30% before its global eradication in 1980 through an intensive vaccination campaign [124,125].

The recent identification of poxviruses in bats emerged from growing scientific interest in their role as potential viral reservoirs [2,126]. To date, bat poxviruses have been detected in species belonging to the *Rousettus*, *Pteropodidae*, and *Vespertilionidae* families [2,126,127], all of which are present in Vietnam [11]. However, despite the confirmed presence of these potential host species, no studies have specifically investigated poxvirus infections in Vietnamese bat populations. Nevertheless, metagenomic analyses of bat samples from southeastern China have detected *Poxviridae* sequences in *Rhinolophus sinicus* populations in Yunnan Province [5,6], a specie that is also widely distributed in Vietnam [11]. However, these studies did not characterize the nature or clinical significance of the poxvirus infections observed [5,6].

The recent identification of Israeli Rousettus aegyptiacus Pox Virus (IsrRAPXV) has significantly expanded our understanding of bat-poxvirus dynamics [128,129]. IsrRAPXV causes high morbidity and mortality in its natural host (*R. aegyptiacus*) [127], representing a rare example of pathogenic poxvirus infection in bats. Furthermore, a documented human case of IsrRAPXV infection [130] demonstrates its possible zoonotic capacity. These findings collectively suggest that the evolutionary relationships and ecological interactions between bats and poxviruses have been substantially underestimated [2,126].

#### 3.2.7. *Togaviridae*

The *Togaviridae* family contains the single genus *Alphavirus*, which includes several medically important human pathogens. These viruses are clinically significant for causing febrile illnesses often accompanied by arthritis and encephalitis. In Asia, chikungunya virus (CHIKV)–transmitted primarily by *Aedes* mosquitoes–represents a major public health concern [131]. CHIKV infection typically presents with high-grade fever, debilitating polyarthralgia, and maculopapular rash, while severe cases may develop neurological complications involving the central nervous system [132,133]. Some patients experience persistent arthralgia that can continue for years post-infection [134,135].

There are serological studies confirming infection of wild bats with alphaviruses, and experimental data indicate their susceptibility to anthropogenic strains [136]. These findings suggest bats could potentially facilitate long-distance viral dispersal [137]. Nevertheless, their precise role in the mosquito-borne transmission cycle of alphaviruses in tropical ecosystems remains poorly characterized [138].

While no studies have specifically investigated *Togaviridae* infections in Vietnamese bats, two Chinese studies conducted in the southeastern border region (adjacent to Myanmar, Laos, and Vietnam) detected multiple human-pathogenic viruses, including members of the *Togaviridae* family [5,6]. This transboundary area represents a potential hotspot for cross-border transmission of bat-borne viruses due to its ecological connectivity and shared bat populations. Specifically, in 2022, metagenomic analysis of *Rhinolophus sinicus* bats in China identified *Togaviridae* viruses, with Chikungunya virus (CHIKV) and Getah virus (GETV) detected in 10% of PCR-positive samples [6]. Both viruses were co-amplified from the same bat specimens, suggesting concurrent circulation of these pathogens in *R. sinicus* populations, and potential for co-transmission within shared geographic habitats. Given that *R. sinicus* is distributed in Vietnam, these findings highlight the need for expanded surveillance of togaviruses in this common reservoir species across Southeast Asia.

### 3.3. Viral Families with Moderate Pandemic Potential

These taxa contain pathogens capable of regional outbreaks with limited human-to-human transmission, as classified by WHO guidelines [33].

#### 3.3.1. *Picornaviridae*

The *Picornaviridae* family represents a diverse group of viruses currently organized into 63 genera and 147 classified species, with numerous additional viruses awaiting taxonomic characterization [139]. Picornavirus infections in humans and animals demonstrate remarkable clinical variability, ranging from asymptomatic cases to severe pathologies affecting multiple organ systems, including the heart (myocarditis), liver (hepatitis), and central nervous system (meningoencephalitis). Transmission of the virus occurs primarily through horizontal spread, via the feco-oral or airborne routes, and through fomites [139]. The *Picornaviridae* family includes several clinically significant human pathogens, notably poliovirus (the causative agent of polio) and hepatitis A virus. In 2024, *Enterovirus coxsackievirus* was added to the WHO’s list of priority pathogens due to its emerging public health threat [33].

To date, bat-associated picornaviruses were identified within the genera *Mischivirus*, *Hepatovirus*, *Crohivirus*, *Kunsagivirus*, *Kobuvirus* and *Shanbavirus* [139]. Phylogenetic analyses reveal that certain bat picornaviruses cluster in monophyletic clades with canine and feline variants [140], suggesting historical cross-species transmission events during the evolutionary history of the *Picornaviridae* family. These findings provide molecular evidence for host-switching behavior among picornaviruses.

Several studies in Vietnam have investigated picornavirus infections in bats and assessed their potential for cross-species transmission [72,73]. This research was conducted under the Vietnam Initiative on Zoonotic Infections (VIZIONS), a comprehensive surveillance program designed to evaluate the zoonotic potential of viruses circulating in rural Vietnamese ecosystems [141]. The initial study focused on characterizing the genetic diversity of kobuviruses and assessing their zoonotic potential across multiple mammalian hosts, including bats [72]. Members of the *Kobuvirus* genus exhibit broad host specificity and are recognized etiological agents of acute gastroenteritis in humans [139]. Within the Vietnamese context, metagenomic analysis of bat guano collected from roosting sites revealed novel kobuvirus strains phylogenetically distinct from known variants. Subsequent host attribution studies identified two reservoir species from the family *Vespertilionidae*: *Scotophilus kuhlii* (*n* = 37 fecal samples) and *Murina ussuriensis* (*n* = 1 fecal sample). In addition, the study established the possibility of transmission of bat-derived kobuvirus to rodents living in the same environment, or vice versa, since the detected bat kobuviruses are closely related to rodent kobuviruses, but direct evidence of virus spread between rodents and bats was not obtained [72]. Phylogenetic analysis reveals that both kobuvirus clades identified in Vietnamese bats demonstrate close evolutionary relationships with Aichivirus A (the prototype human kobuvirus), suggesting potential historical zoonotic connections between bat and human strains [72].

As part of the ongoing VIZIONS program, a subsequent large-scale study characterized *Picornaviridae* diversity across 2100 samples, including 179 bat fecal specimens [73]. Among bats, in addition to the previously described genus *Kobuvirus*, viruses belonging to the genus *Parechovirus* were identified. The bat parechovirus sequences belong to one new species [73]. This investigation revealed predominant *Picornaviridae* prevalence in bats (67% detection rate), with frequent co-infections (57% of positive samples). All identified bat picornavirus sequences constituted previously undescribed genetic variants, including first detection of parechoviruses in bats, representing a novel species within the genus *Parechovirus*, expanding known viral diversity in this host group [72,73].

Complementing the Vietnamese bat picornavirus studies, multiple metagenomic analyses of bats in southern China have yielded concordant findings [5,6,7,8]. Virome characterization data from these Chinese bat populations demonstrate consistency with Vietnamese results regarding both *Picornaviridae* infection prevalence and genetic diversity [72,73], suggesting similar patterns of picornavirus circulation across the broader Southeast Asian region.

Investigating viral evolutionary history and host–virus coevolution patterns provides critical insights for predicting emerging zoonotic diseases [142]. Picornaviruses demonstrate remarkable ecological flexibility, infecting diverse bat genera and demonstrating cross-species transmission capacity among bat populations [72,143]. Phylogenetic evidence further indicates that documented cases of interspecies transmission likely represent only a fraction of actual cross-species spillover events, suggesting substantial underestimation of their evolutionary potential [72,73,144,145].

#### 3.3.2. *Retroviridae*

The *Retroviridae* family includes two subfamilies encompassing eleven genera. Retroviruses are widespread in nature, occurring in vertebrate hosts. Retroviruses exhibit broad distribution among vertebrate hosts, with varying pathogenic potential [146,147]. While many retroviruses are non-pathogenic, human infections can lead to severe clinical outcomes including immunodeficiency (e.g., HIV-1 and HIV-2 causing AIDS) and oncogenesis (e.g., HTLV-1 associated with adult T-cell leukemia). Transmission occurs through multiple routes through exposure to infected biological fluids [148].

While systematic screening of Vietnamese chiroptera for retroviruses has not been conducted, multiple studies in neighboring countries have documented bat retrovirus infections. A Chinese surveillance study initially designed to detect paramyxoviruses in bat swabs unexpectedly identified several retroviruses, including classified viruses from the *Gammaretrovirus* and *Betaretrovirus* genera as well as novel unclassified bat retroviruses [66]. Phylogenetic analysis of *Pol*, *Gag*, and *Env* protein-coding regions revealed distinct evolutionary patterns, with the *Env* region demonstrating significantly lower sequence homology to known bat gammaretroviruses compared to the more conserved Pol and Gag regions. This notable divergence in the envelope glycoprotein suggests either accelerated evolution under host immune pressure, or recombination events with distantly related retroviruses. Such genetic plasticity in the *Env* region may facilitate the emergence of novel viral variants with altered host tropism [66].

Two additional metagenomic studies of Chinese bat viromes reported high prevalence rates of retroviral sequences, though without further molecular characterization [5,6]. Nevertheless, the bat species sampled in these studies (*Rhinolophus* spp. *and Hipposideros* spp.) are also distributed throughout Vietnam [11], suggesting potential regional circulation of these retroviruses.

#### 3.3.3. *Adenoviridae*

Members of the *Adenoviridae* family are among the most common viruses infecting vertebrates, ranging from fish to humans [149]. They cause respiratory, gastrointestinal, and ocular infections of varying severity [150]. Although adenoviruses typically demonstrate strict host specificity, documented cross-species transmission events (e.g., human adenovirus group C infections in gorillas [151]) underscore their evolutionary plasticity and zoonotic potential.

While adenovirus surveillance data from Vietnamese bats remain limited, multiple studies in neighboring countries have confirmed active adenovirus circulation in several local bat species such as *Rhinolophus pusillus*, *Scotophilus kuhlii*, etc. [5,7,74]. Metagenomic analysis of 235 bats in southeastern China identified mastadenoviruses in *Rhinolophus pusillus* [5]. A complementary study of 149 bats from Yunnan Province (bordering Vietnam) detected nine adenovirus species in rectal samples from *Hipposideros larvatus* and *Cynopterus sphinx* [7]. Nine adenovirus species were detected in *Hipposideros larvatus* and *Cynopterus sphinx*. Chinese researchers have isolated the bat adenovirus TJM–phylogenetically clustered with canine adenoviruses–showing an 8% prevalence across several species including *Scotophilus kuhlii* and *Hipposideros armiger* [74]. All these reservoir species (*Rhinolophus* spp., *Hipposideros* spp., *Cynopterus* spp., and *Scotophilus* spp.) are established components of Vietnam’s chiropteran fauna [11], suggesting potential undetected adenovirus circulation in the region.

Thus, modern adenoviruses, despite coevolutionary adaptations to hosts, demonstrate remarkable evolutionary plasticity, maintaining zoonotic potential despite extensive host-adapted evolution [151,152]. Their high transmissibility, resistance of the viral particle to external factors [153] and efficient propagation in high-density populations through respiratory droplets, fomites, and fecal-oral routes [154] facilitate rapid community spread and sustained transmission chains, particularly in congregate settings.

### 3.4. Viral Families with Low Pandemic Potential

These taxa show minimal evidence of human spillover or severe disease, per WHO criteria of public health emergencies of international concern (PHEIC) [33].

#### 3.4.1. *Astroviridae*

The *Astroviridae* family, comprising mammalian (*Mamastrovirus*) and avian (*Avastrovirus*) lineages, demonstrates remarkable adaptive potential through two key evolutionary mechanisms. First, their exceptional genetic plasticity manifests as high mutation rates from error-prone replication combined with frequent recombination events between circulating strains [78,155].

Surveillance studies of bats in Vietnam’s neighboring countries (Cambodia and Laos) detected astroviruses in approximately 5% of samples, with particularly high prevalence rates (about 42%) observed in certain genera such as *Myotis* Phylogenetic analysis revealed that bat astroviruses clustered closely with strains isolated from ungulates and porcupines, providing genomic evidence of cross-species transmission events among these mammalian hosts [78]. Studies of astroviruses in Chinese bat populations, including 19 species also found in Vietnam, further support their role as key natural reservoirs of viral genetic diversity [5,7,8,55,75,76,77,156]. These findings highlight the extensive astrovirus circulation in bats across Southeast Asia, with implications for regional host switch risk assessment.

Recent studies in Southeast China have documented exceptionally high prevalence rates of astroviruses, reaching 100% in *Miniopterus magnater* populations [75]; showed substantial genetic divergence from known reference sequences, with as little as 66.6% identity score [7]; revealed phylogenetic evidence of cross-species transmission events between sympatric bat species, including viral sharing between *M. magnater* and *M. pusillus* [75]. These findings collectively underscore the dynamic evolution and interspecies mobility of astroviruses in regional bat populations.

In addition, studies have identified frequent coinfection events involving multiple astrovirus strains [75], with 62% of bats simultaneously harboring viruses from different families [5]. Viral coinfections create a permissive environment for genetic recombination and elevate cross-species transmission risks by producing viral lineages exhibiting altered receptor binding affinity or modified tissue tropism.

The obtained data demonstrate ongoing evolution and adaptation of astroviruses in bat populations, with recombination events and coinfections facilitating the generation of novel viral variants. The observed high genetic diversity, even at the intraspecies level, underscores bats’ role as important astrovirus reservoirs. These frequent recombination events highlight the need for strengthened epidemiological surveillance, particularly in Southeast Asia where human-bat interfaces are common.

#### 3.4.2. *Rhabdoviridae*

The *Rhabdoviridae* family represents one of the most ecologically diverse viral families, currently comprising four subfamilies, 56 genera, and 434 formally recognized species [157]. These viruses exhibit an exceptionally broad host range, infects animals, plants, and arthropods [158]. Key human pathogens include rabies virus (fatal encephalitis) [159,160], Bas-Congo (hemorrhagic fever) [161], and Chandipura (encephalitis) [162]. Current taxonomic classifications recognize at least 15 distinct Lyssavirus species capable of causing rabies-like encephalitis [157] and almost all of them have been found in bats [163,164,165], indicating bats as the primary evolutionary reservoir of lyssaviruses [166]. Beyond Lyssavirus species, bats also harbor rhabdoviruses from two additional genera, *Vesiculovirus* and *Ledantevirus* [82,164,167,168]. Ledanteviruses exhibit a global distribution in bat populations, with documented sporadic human infections. However, their pathogenic potential in humans and precise transmission mechanisms remain poorly characterized [169,170,171]. *Vesiculoviruses* belong to the arbovirus transmitted primarily through insect vectors [131,157]. While serological evidence confirms their ability to infect diverse vertebrate hosts in endemic regions, key aspects of their natural transmission cycle remain poorly characterized, particularly for mammalian hosts [157].

Recent Chinese studies have substantially advanced our comprehension of bat-associated rhabdovirus diversity, uncovering multiple novel genetic lineages with zoonotic potential. In 2022, researchers identified Rhinolophus rhabdovirus DPuer in *Rhinolophus affinis* bats [83]–a species also distributed in Vietnam [11]. Preliminary characterization suggests this virus possesses broad host tropism, including potential zoonotic capacity [83]. A separate investigation discovered three novel rhabdoviruses [82], two of which (isolated from *Rhinolophus* spp.) may represent new *Vesiculovirus* species based on phylogenetic analysis.

While rhabdoviruses are currently classified as having limited pandemic potential, their demonstrated zoonotic capacity and high case fatality rates in human infections warrant serious concern highlighting the critical need for systematic surveillance for early detection of emerging rhabdovirus threats.

#### 3.4.3. *Parvoviridae*

The *Parvoviridae* family is divided into two subfamilies, with only *Parvovirinae* members infecting vertebrate hosts. Current taxonomy recognizes eight monophyletic genera within *Parvovirinae*, five of which include known human pathogens [172]. Parvovirus transmission occurs primarily through the feco-oral route. While parvoviruses can elicit diverse clinical manifestations ranging from mild erythema infectious to severe manifestations in immunocompromised patients, most infections in immunocompetent individuals are self-limiting and non-life-threatening [173]. This stands in stark contrast to the severe pathogenicity observed in carnivore-infecting parvoviruses. Canine parvovirus (CPV), which emerged in the 1970s through cross-species transmission of feline panleukopenia virus [174], demonstrates particularly high virulence with fatality rates approaching 90% [175].

While bats constitute a major reservoir host for numerous emerging viruses, documented parvovirus infections in chiropteran species remain relatively rare. However, recent findings indicate potential cross-species transmission of *Bocaparvovirus* between bats and swine populations, suggesting zoonotic capacity [176], and intraspecific circulation of diverse parvovirus strains within bat colonies [176,177].

While systematic surveillance has yet to detect *Parvoviridae* family members in Vietnamese bats, their likely presence is suggested by documented parvovirus infections in local bat species across the Chinese border [5,6,7,8,84,85]. A targeted investigation of *Dependoparvovirus* diversity in Chinese bats analyzed 370 samples across 19 chiropteran species [84], The study documented viral presence in seven microchiropteran species that exhibit transboundary distributions in both China and Vietnam [11]: *Rhinolophus affinis*, *Rhinolophus sinicus*, *Rhinolophus pearsoni*, *Rhinolophus macrotis*, *Hipposideros armiger*, *Hipposideros larvatus*, *Myotis ricketti*. These findings demonstrate widespread circulation of bat adeno-associated viruses (*Dependoparvovirus*) across diverse Asian bat populations. This conclusion is further supported by multiple independent metagenomic surveys in China [5,6,7,8], which have consistently establish bats as significant reservoirs of parvoviral diversity in the region, with important implications for cross-species transmission risk assessment.

#### 3.4.4. *Spinareoviridae* and *Sedoreoviridae*

The order *Reovirales* currently comprises two distinct families, *Spinareoviridae* and *Sedoreoviridae* [178,179], divided based on structural characteristics. Pathogens have a wide host range, infecting mammals, fish, birds, reptiles, arthropods, algae, fungi and plants. The families include 15 genera, nine genera within *Spinareoviridae* and six for *Sedoreoviridae* [178,179]. The *Spinareoviridae* family includes several mammalian pathogens of clinical importance, notably *Coltivirus*, which causes Colorado tick fever [180], and *Orthoreovirus*, associated with respiratory and enteric infections in humans [181,182]. Similarly, the *Sedoreoviridae* family contains multiple medically relevant genera, including *Orbivirus* (encompassing veterinary pathogens like bluetongue virus with zoonotic potential) [183,184], *Rotavirus* (a major global cause of severe pediatric gastroenteritis) [185], and the emerging mosquito-borne *Seadornavirus* [186]. In Europe, a relationship has been established between mammalian orthoreoviruses found in European bats and one isolated from a child admitted to hospital with acute gastroenteritis [187]. Also, based on epidemiological data, it is believed that the Malacca and Kampar viruses, the causative agents of acute respiratory infections in humans in Malaysia, originated from bats [188]. *Spinareoviridae* and *Sedoreoviridae*, while sharing the segmented dsRNA genome characteristic of *Reovirales*, demonstrate distinct clinical manifestations and epidemiological patterns that reflect their structural and biological differences.

Due to the recent taxonomic reclassification that divided the former *Reoviridae* family into *Spinareoviridae* and *Sedoreoviridae* [189], it remains methodologically challenging to retrospectively reclassify virome data from bats in Vietnam and adjacent regions according to current taxonomy. Therefore, we have maintained the previous classification scheme when analyzing historical bat virome data from Vietnam and neighboring regions.

Virome analyses of Vietnamese bat populations have demonstrated widespread circulation of reoviruses across multiple species [5,6,7,8,86,87]. This observation is supported by a 2023 Chinese metagenomic surveillance study, which identified *Reovirales* as the predominant viral order, detected in 27.5% of virus-positive samples [7]. Molecular surveillance has identified distinct bat rotavirus strains circulating among specific chiropteran hosts in the region. Bat rotavirus type CX1 was detected in *Aselliscus stoliczkanus* (Stoliczka’s trident bat) and multiple *Rhinolophus* species (horseshoe bats) [7]. Meanwhile, rotavirus types WD1 and WD2 were found associated with *Eonycteris* (dawn bats) and *Rousettus* (rousette bats) species, respectively [7]. Both genera are widely distributed throughout Vietnam [11]. In an earlier study conducted between 2007 and 2012, researchers identified six novel reoviruses circulating in *Hipposideros* (leaf-nosed bats) and *Myotis* (mouse-eared bats) populations. Phylogenetic characterization revealed these bat-derived strains formed a monophyletic clade with known mammalian orthoreoviruses, demonstrating closest evolutionary relationships to porcine and mustelid (mink) orthoreovirus lineages [87].

The findings demonstrate that multiple ecological groups of Vietnamese bats maintain natural reservoirs of reovirus infections [7,86]. Of particular epidemiological significance is the identification of strains closely related to those circulating in domestic animals and human populations [86,87], suggesting potential interspecies transmission at human–animal interfaces. These results underscore the importance of sustained pathogen surveillance in the region, particularly considering Vietnam’s diverse and widespread bat populations.

#### 3.4.5. *Orthoherpesviridae* (*Herpesviridae*)

Members of the order *Orthoherpesvirales* exhibit a long evolutionary history of host co-adaptation, with species-specific variants likely existing in nearly all mammalian, avian, and reptilian species [190]. Following initial infection, these viruses establish lifelong latency through sophisticated mechanisms including limited viral gene expression and periodic reactivation. In human hosts, severe disease manifestations primarily occur in three vulnerable populations such as immunocompromised individuals, neonates with immature immune systems or in utero [190].

Recent virological surveys in Southeast Asia have significantly expanded our knowledge of bat-associated herpesviruses. In Vietnam, molecular analysis of biological samples (10 urine and 206 fecal specimens) from *Pteropus lylei* (Lyle’s flying fox) identified a novel *Alphaherpesvirus* species [88]. Parallel research in China detected previously unknown *Betaherpesvirus* and *Gammaherpesvirus* lineages in two cave-dwelling species: *Myotis davidii* (David’s myotis) and *Rhinolophus pusillus* (least horseshoe bat) [89]. Of particular relevance, *R. pusillus* maintains a transboundary distribution that includes both China and Vietnam [11], suggesting potential for cross-border viral transmission.

#### 3.4.6. *Hepeviridae*

Bat-associated hepeviruses, classified within the genus *Chirohepevirus*, exhibit a global distribution across diverse chiropteran species, including both insectivorous bats and frugivorous pteropodids [90,191]. Genomic analyses of bat hepeviruses reveal these viruses represent relatively recent host-switching events into mammalian reservoirs, but no zoonotic transmission of *Chirohepevirus* to humans has been documented to date [191]. However, the species *Paslahepevirus balayani* and *Rocahepevirus ratti* of the related genus *Paslahepevirus*, which are clinically significant human pathogens capable of causing hepatitis, glomerulonephritis, pancreatitis, and neurological manifestations [192].

Hepeviruses are primarily transmitted via the fecal-oral route, predominantly through ingestion of contaminated water or consumption of undercooked meat products [192]. However, experimental studies demonstrating successful cross-species transmission of swine hepeviruses to non-human primate models provide compelling evidence of their ability to overcome species barriers [193,194]. These findings underscore the zoonotic potential of certain hepevirus lineages.

While a Vietnamese study examining bat *Hepeviridae* infections reported no positive detections [91], several studies in China have documented these viruses in chiropteran hosts [5,6], identifying *Hepeviridae* sequences in bat samples. Though these findings were not fully characterized at the genomic or epidemiological level.

While bat-associated *Chirohepeviruses* have not been documented in human infections to date, the demonstrated capacity of other hepeviruses to cross species boundaries suggests the need for ongoing surveillance of emerging strains across animal reservoirs. These findings suggest bats may serve as significant reservoirs of *Hepeviridae*, particularly given their habitat overlap with human populations and the underexplored zoonotic potential of these viruses. However, the distribution and prevalence of hepeviruses in Vietnamese bat populations remain poorly characterized.

#### 3.4.7. *Papillomaviridae*

Papillomaviruses, which exhibit a tropism for epithelial cells, have been identified across diverse vertebrate taxa, including multiple bat species [2,195]. Although these viruses typically demonstrate high host specificity [196], a series of molecular evidence suggests they possess underappreciated capacity for cross-species transmission [197,198,199]. Recent years have seen a marked increase in both surveillance studies documenting novel bat papillomaviruses [200], and phylogenetic analyses revealing interspecies transmission events among chiropteran hosts [201,202]. Of particular concern, a documented case of basal squamous cell carcinoma in *Rousettus aegyptiacus* (Egyptian fruit bat) was associated with bovine papillomavirus infection [203] representing an evidence of cross-mammalian species viral transmission.

Metagenomic analyses of Chinese bat populations in provinces bordering Vietnam have identified papillomavirus infections across multiple species [5,6,8] including *Rhinolophus affinis*–a common for species Vietnam species [11], which harbored papillomavirus sequences assigned to the novel *Dyosigmapapillomavirus* [8].

#### 3.4.8. *Polyomaviridae*

The *Polyomaviridae* family demonstrates broad host range across vertebrates, arthropods (including insects and arachnids) [204]. Polyomaviruses generally maintain strict host specificity in mammalian species [205,206], but their transmission dynamics remain poorly characterized for most host systems [204]. While interspecies transmission events resulting in productive polyomavirus infection remain uncommon certain members of the *Polyomaviridae* family are established pathogens in their natural hosts [204,207]. Current taxonomy recognizes six genera within the family [204], among them, viruses belonging to the *Alphapolyomavirus* and *Betapolyomavirus* genera have been identified in various bat species [208,209]. Over the past decade, 23 new polyomavirus species have been isolated from bats and recognized by ICTV [209].

While no systematic investigations of polyomaviruses in Vietnamese bats have been conducted to date, multiple studies from neighboring Yunnan Province, China, have documented the presence of *Polyomaviridae* family members in regional chiropteran populations [7,66]. A 2014 surveillance study employed a two-phase approach to investigate paramyxovirus infections in bats, subsequently utilizing high-throughput sequencing to characterize coinfections in positive samples [66]. From approximately 7000 assembled contigs, genomic analysis identified three polyomavirus sequences showing significant homology to known polyomaviruses: hamster polyomavirus (Mesocricetus auratus polyomavirus 1), Merkel cell polyomavirus (Human polyomavirus 5), and STL polyomavirus (Human polyomavirus 12) [66]. These findings demonstrate evolutionary connections between bat and mammalian (including human) polyomavirus lineages. Another study have identified Bat polyomavirus CX1 in two horseshoe bat species (*Rhinolophus pusillus* and *Rhinolophus stheno*) [7] that maintain geographic ranges extending into Vietnam [11], showing evidence of both host-specific adaptation and potential cross-species transmission events.

The combination of widespread host distribution and unresolved transmission ecology makes polyomaviruses an important yet understudied group in viral evolution research.

#### 3.4.9. *Hepadnaviridae*

Hepadnaviruses exhibit strict hepatotropism, capable of establishing either transient or persistent infections in their hosts. Among these, *Orthohepadnavirus* species–particularly human hepatitis B virus (HBV)–pose significant public health threats [210].

Genomic studies have identified hepadnavirus sequences in five bat species across diverse geographic regions, including Panama, Gabon, Myanmar, and China [211]. Of particular relevance to Vietnam’s viral ecology are findings from southern China, where in a 2008–2013 study, researchers analyzed 78 liver tissue samples collected from 17 bat species [90]. Viral screening identified four hepadnavirus-positive specimens, with two detections each in *Rhinolophus sinicus* and *Rhinolophus affinis*. Phylogenetic characterization revealed these isolates formed a distinct clade with *Hipposideros pomona* (Pomona leaf-nosed bat) hepadnaviruses previously identified in Pu’er City, Yunnan Province (2011) [212]. These findings demonstrate strong evolutionary relationships among Asian bat hepadnaviruses as well as potential biogeographic patterning in hepadnavirus evolution.

Molecular screening of liver samples from bats and rodents revealed distinct patterns of hepatitis virus prevalence: hepatitis B-like sequences were detected in 1.9% (3/157) of bats, while hepatitis C and E homologs were found in 8.1% (38/470) and 3% (14/470) of rodents, respectively [91]. The bat-associated hepatitis B sequences, identified in *Hipposideros pomona* and *Hipposideros larvatus*, demonstrated substantial genetic conservation (85–92% nucleotide identity) with previously characterized viral strains. The findings underscore the importance of continued genomic surveillance to monitor viral evolution and transmission dynamics in these animal reservoirs.

### 3.5. Viral Families with No Demonstrated Pandemic Potential

Virus families with no members with risk of causing a public health emergency of international concern [33].

#### 3.5.1. *Circoviridae*

The *Circoviridae* family includs the smallest known animal viruses. Current taxonomy divides this family into two genera: *Circovirus* and *Cyclovirus*, which exhibit distinct host ranges and biological characteristics [213]. *Circovirus* are found exclusively in vertebrates [213], and include several economically significant pathogens affecting swine (Porcine circovirus), poultry (Beak and feather disease virus), and other domesticated animals [214,215]. In contrast, *Cyclovirus* species show broader host associations, having been identified in both vertebrate and invertebrate hosts (particularly arthropods), though their natural reservoirs and transmission cycles remain poorly characterized [213].

Since their initial discovery in bats during the early 2000s [216,217], circoviruses have been increasingly recognized as widespread constituents of the global bat virome, with over 30 distinct *Circoviridae* species currently documented across Asian, American, and European bat populations [213]. A 2018 molecular characterization study identified two novel bat-associated Circovirus species distinguished by a unique poly-T motif within their intergenic regions [93]. Screening of 79 specimens from two ecologically important bat species–*Vespertilio sinensis* (Chinese pipistrelle, endemic to China) and *Hipposideros armiger* (great roundleaf bat, distributed across China and Vietnam)–revealed a high prevalence rate (41.8%, 33/79 positive samples). While the study did not stratify infection rates by host species, the inclusion of *H. armiger* (a known Vietnamese species) suggests potential for cross-border viral circulation given this species’ extensive range throughout Southeast Asia [11].

While circovirus detections frequently occur as incidental findings during broader bat virome studies [5,6,7,8], a comprehensive multinational investigation has provided systematic data on their global distribution. This large-scale study examined over 80 bat species across diverse ecosystems in eight countries spanning four continents, representing one of the most extensive surveys of bat-associated viruses to date [94]. The study confirmed active circovirus circulation among bat populations in Southeast Asia, with notable detections in Vietnam despite limited sampling (19 species). Three bat species tested positive: *Hipposideros armiger*, *Rhinolophus affinis*, and *Phoniscus* sp. Remarkably, the observed detection rate in Vietnamese bats aligned with prevalence estimates from more extensively sampled regions [94].

Although the zoonotic potential of bat-associated circoviruses remains uncharacterized, their close relationship to terrestrial mammalian circoviruses [93] and possible cross-species transmission events among vertebrate hosts [218,219] highlight the need for further monitoring of these viral group, especially in Southeast Asia due to a high frequency of contacts between bats, domestic animals and humans in the region [156].

#### 3.5.2. *Caliciviridae*

The *Caliciviridae* family includes 11 genera, 7 of which affect mammals [220]. Human noroviruses are the main cause of acute gastroenteritis, usually with a mild course, but with a risk of mortality in the elderly and immunocompromised individuals [221]. Members of the *Caliciviridae* family have been detected in multiple bat species across Asia, with genomic analyses revealing significant phylogenetic relationships between these bat-associated strains and known human caliciviruses [5,97,222].

While no direct studies have investigated caliciviruses in Vietnamese bats, compelling indirect evidence suggests their circulation throughout the region. Most notably, a 2021 metagenomic study in China’s Yunnan Province (bordering Vietnam) identified a novel norovirus strain in *Rhinolophus pusillus* bats that exhibited Sufficient genetic divergence (per ICTV guidelines) to qualify it as a new species [5]. Virome analysis of *Rh. affinis* from Hainan Island, China, revealed *Caliciviridae* sequences among 17 viral families [8], highlighting the wide viral diversity in *Rhinolophus* representatives. Sapoviruses genetically related to caliciviruses of non-human primates [7] were detected in *Cynopterus sphinx*, which is also distributed in Vietnam [11]. The observed phenomena can be explained by overlapping patterns of carbohydrate receptor binding site in bat and human caliciviruses indicating the ability to overcome interspecies barriers [7,222].

#### 3.5.3. Other Taxonomic Groups

Virome studies routinely detect diet-derived and microbiome-associated viruses in bats [6,7], yet research efforts remain mainly focused on mammalian viruses while neglecting food-borne viral constituents. In this review article, we have also prioritized viral families with established zoonotic potential or association with mammalian host-species to better characterize possible public health risks.

Virome studies in Vietnamese bats have identified several mammalian-associated virus families (*Anelloviridae*, *Picobirnaviridae*, *Asfarviridae* [5,7,8]), though their human pathogenicity remains unconfirmed. These studies have revealed unique viral findings including unclassified basaviruses (order *Picornavirales*) [96] and Kaeng Khoi orthobunyavirus (order *Bunyavirales*) [95], whose zoonotic potential warrants further investigation. Of particular concern is the detection of Kaeng Khoi virus in cave systems with high tourist visitation rates raises significant public health concerns regarding potential human exposure and zoonotic transmission. While current evidence suggests these viruses pose minimal risk to humans, their persistent circulation in bat populations underscores the importance of ongoing surveillance to monitor potential emergence.

## 4. Summary

This review identifies distinct epidemiological patterns for bat-borne viruses in Vietnam associated with Public Health Emergencies of International Concern (PHEIC). Surveillance of bat-borne viruses in Vietnam reveals a distinct regional profile compared to other parts of Southeast Asia with infection rates for most high-risk viral families being relatively low, with a notable exception of coronaviruses.

The *Coronaviridae* family was not only the most frequently detected across all studies but also the only family present in every region examined. The overall coronavirus infection rate in Vietnamese bats, based on aggregated data from the past 23 years, is 30%–significantly higher than in neighboring China (10%), Cambodia (5%), and Laos (3.5%). This elevated and dynamic prevalence, which peaked at 67.6% in a 2013–2014 cohort [27], underscores a unique regional pattern and highlights the critical need for sustained, targeted monitoring of coronaviruses in Vietnamese bat populations.

Infection rates for other high-risk PHEIC agents in Vietnam are low or undetectable. Filovirus prevalence is approximately 1%, markedly lower than the 18% reported in China [67]. While earlier studies did not detect filoviruses in Vietnamese bats [26], recent molecular evidence has identified filovirus RNA in *Rousettus leschenaultii* and *R. amplexicaudatus* in northern Vietnam [71], suggesting dynamic shifts in viral circulation. Similarly, hantavirus infection rates are 2.4% in Vietnam compared to 8.4% in China. Notably, members of the *Flaviviridae*, *Poxviridae*, and *Togaviridae* families were not detected in Vietnamese bats, whereas Chinese studies reported their presence only in associated metadata [5,6,8], although there were studies in Vietnam in which they looked for *Flaviviridae* [28,91]. A parallel trend is observed with coronaviruses, the most extensively studied viral family in the region over the past five years. This contrasting profile, characterized by high coronavirus prevalence alongside low or undetectable rates of other highly pathogenic families, may reflect genuine country-specific viral circulation dynamics or, alternatively, methodological differences in surveillance and sample collection between the studied regions.

The broader diversity of viral families detected in southern provinces of China reasonably suggests that comparable viral diversity likely exists in Vietnamese bat populations, awaiting discovery through expanded surveillance efforts. Priority in future investigations should be given to the surveillance for viral families such as *Adenoviridae*, *Astroviridae*, *Caliciviridae*, *Hepeviridae*, *Papillomaviridae*, *Parvoviridae*, *Picobirnaviridae*, *Polyomaviridae*, *Spinareoviridae*, *Sedoreoviridae*, and *Retroviridae*. These families are frequently detected in bats, but are missing from the current dataset for Vietnam. The detection of *Astroviridae* is particularly important because these viruses are widespread among many genera of bats native to Vietnam and have been detected in all neighboring regions except Vietnam itself.

The evidence systematized in this review is subject to several limitations. First, the restriction of the literature search to PubMed and English-language publications, combined with uneven geographical and taxonomic sampling coverage, introduces a potential for selection bias and false-negative findings, thereby limiting the comprehensiveness of our results. Additionally, the omission of a formal risk of bias assessment means that the influence of varying study methodologies on the reported detections remains unquantified. Consequently, heterogeneity in diagnostic sensitivity, sampling intensity, and data reporting likely resulted in a substantial underestimation of true viral diversity. Consequently, the current understanding of Vietnam’s bat virome is constrained by a limited number of studies and a pronounced research bias towards specific high-priority viral families like *Coronaviridae* and *Filoviridae*. To achieve a more representative profile, future research should employ agnostic methods, such as metagenomic sequencing or multiplex PCR, capable of simultaneously detecting a broad range of viral families within a standardized sample pool. Overall, these findings underscore the necessity of systematic and cross-border virome surveillance in Vietnam to better account for the diversity and zoonotic potential of bat-associated viruses in the region.

## 5. Conclusions

This review characterizes the bat virome of Vietnam, with a focus on its zoonotic potential and cross-border transmission risks. We identified viruses from 32 families, 29 of which have known public health relevance. Despite hosting one of the world’s most diverse bat faunas, Vietnam’s bat virome remains relatively understudied. The broader diversity of viral families detected in neighboring countries suggests that a similar yet underexplored viral diversity likely exists in Vietnamese bat populations, awaiting discovery through expanded and systematic surveillance efforts–with at least 13 viral families, including *Astroviridae*, yet to be detected.

The documented viral diversity observed in Vietnamese bats mirrors global virome patterns, reinforcing their established role as reservoir hosts for several viral families with particular importance, including *Coronaviridae*, *Filoviridae*, and *Paramyxoviridae*. The described distribution of viral families and relationships between bat families and viruses highlights the importance of monitoring specific viral families in specific bat populations, e.g., *Filoviridae* are related to *Pteropodidae*, while *Rhabdoviridae* and *Hantaviridae* are widely distributed among bat populations.

These findings collectively underscore the critical need for expanded virome surveillance in Vietnamese bats populations. Such efforts should aim to: (1) systematically characterize viral diversity which enhanced taxonomic resolution, (2) identify potentially pathogenic viruses with zoonotic potential, and (3) develop evidence-based strategies for epidemic prevention. To achieve these goals, future studies should implement both systematic sampling across broader geographic ranges in Southeast Asia, and advanced genomic characterization methods to achieve higher taxonomic resolution. Additionally, the representativeness of the data obtained about the bat virome of Vietnam will significantly benefit from the research utilizing an agnostic rather than single-family focused approach, including the metagenomic sequencing or multiplex screening, targeting a broad range of viral families simultaneously with a unified methodology within a sample pool. This integrated strategy will provide a more comprehensive characterization of bat virome diversity, enhanced capacity to monitor viral evolutionary trajectories, and improved risk assessment of potential zoonotic transmission in the region.

This review reveals a significant, yet understudied viral diversity in Vietnamese bats, with clear ecological connections to neighboring regions. The detection of multiple virus families designated by WHO (2024) [33] as high-priority pathogens highlights the significant zoonotic potential of local bat populations. The observed host–virus associations align with global patterns, suggesting that the drivers of these relationships are widespread. Strengthening systematic surveillance programs that combine molecular diagnostics, metagenomic sequencing, and cross-border data integration is essential. Such multidimensional data will be critical for developing proactive surveillance systems and predictive models of emergence of bat-borne pathogens in Southeast Asia.

## Figures and Tables

**Table 1:** Virome composition of bats in Vietnam: taxonomic classification, host range, and zoonotic risk assessment. Virus families were categorized according to their epidemiological risk and zoonotic potential using the WHO priority pathogen framework (2024) [33]. Each family was assigned to the highest-risk category represented by any of its members.

Viral Family	Genome	Host	Publication Number	References
**High risk**				
*Coronaviridae*	(+)RNA	Mammals, birds, amphibians, fish	21	[5,7,8,25,26,27,28,49,50,51,52,53,54,55,56,57,58,59,60,61,62]
*Paramyxoviridae*	(−)RNA	Mammals, birds, reptiles, fish	9	[7,8,26,28,63,64,65,66,67,68]
*Hantaviridae*	(−)RNA	Mammals, reptiles, fish	5	[29,30,31,32,69]
*Filoviridae*	(−)RNA	Mammals, reptiles, fish	3	[67,70,71]
*Flaviviridae*	(+)RNA	Mammals; most *Orthoflavivirus* are arthropod-borne	3	[5,6,8]
*Poxviridae*	dsDNA	Vertebrates, arthropods	3	[5,6,8]
*Togaviridae*	(+)RNA	Vertebrates, arthropods; most alphaviruses are mosquito-borne	2	[5,6]
**Medium risk**				
*Picornaviridae*	(+)RNA	Vertebrates	6	[5,6,7,8,72,73]
*Retroviridae*	ssRNA-RT	Mammals, birds, signs of ERVs in genomes of other vertebrates and invertebrates	4	[5,6,8,66]
* *Adenoviridae*	dsDNA	Vertebrates	3	[5,7,74]
**Low risk**				
*Astroviridae*	(+)RNA	Birds, Mammals	8	[5,7,8,55,75,76,77,78]
*Rhabdoviridae*	(−)RNA	Vertebrates, insects and plants; many vertebrate and plant rhabdoviruses are arthropod-borne	7	[7,67,79,80,81,82,83]
*Parvoviridae*	dsDNA	Mammals, birds, reptiles, insects, crustacea, echinoderms	6	[5,6,7,8,84,85]
*Spinareoviridae and* *Sedoreoviridae*	dsDNA	Mammals, fish, birds, reptiles, arthropods, plants, fungi	6	[5,6,7,8,86,87]
*Orthoherpesviridae*	dsDNA	Mammals, birds, reptiles	5	[5,6,8,88,89]
*Hepeviridae*	(+)RNA	Mammals, birds, fish	3	[5,6,8]
*Papillomaviridae*	dsDNA	Mammals, birds, reptiles, fish	3	[5,6,8]
*Polyomaviridae*	dsDNA	Mammals, birds, fish	3	[7,8,66]
*Hepadnaviridae*	dsDNA	Vertebrates	2	[90,91]
*Anelloviridae*	ssDNA	Mammals, birds	2	[7,8]
*Picobirnaviridae*	dsDNA	Mammals, birds, reptiles, invertebrates	1	[8]
**Other**				
*Circoviridae*	ssDNA	Mammals, birds, fish, arthropods	6	[5,6,7,8,92,93,94]
*Caliciviridae*	(+)RNA	Mammals, birds, fish	3	[5,7,8]
Unclassified Order: *Picornavirales*, Class: *Bunyaviricetes*	RNA	Vertebrates, invertebrates, protists, plants	2	[95,96]
*Asfarviridae*	dsDNA	Suidae	1	[5]

* high-medium risk according to WHO.

## Data Availability

Not applicable.

## Supplementary Materials

The following supporting information can be downloaded at: https://www.mdpi.com/article/10.3390/v17121532/s1. Table S1: Characteristics of studies included in the review of bat-associated viruses in Vietnam (2002–2024): host species coverage, detected viral families, and prevalence rates. Table S2: Regional distribution of bat-associated viruses in Vietnam and neighboring territories: geographic occurrence, taxonomic classification, and surveillance intensity (2002–2024). Table S3: Transmission patterns of bat-associated viruses in Southeast Asia: regional distribution and host–pathogen network analysis (2002–2024). Figure S1: PRISMA flow diagram.
