# Supplementary material for: Characterizing the Bat Virome of Vietnam: A Systematic Review of Viral Diversity and Zoonotic Potential
Source: Viruses. 2025 Nov 22;17(12):1532. doi: 10.3390/v17121532 (PMC12737739; doi:10.3390/v17121532)
Supplement: Supplementary file 1 [file viruses-17-01532-s001.zip › PRISMA_2020_flow_diagram.pdf]

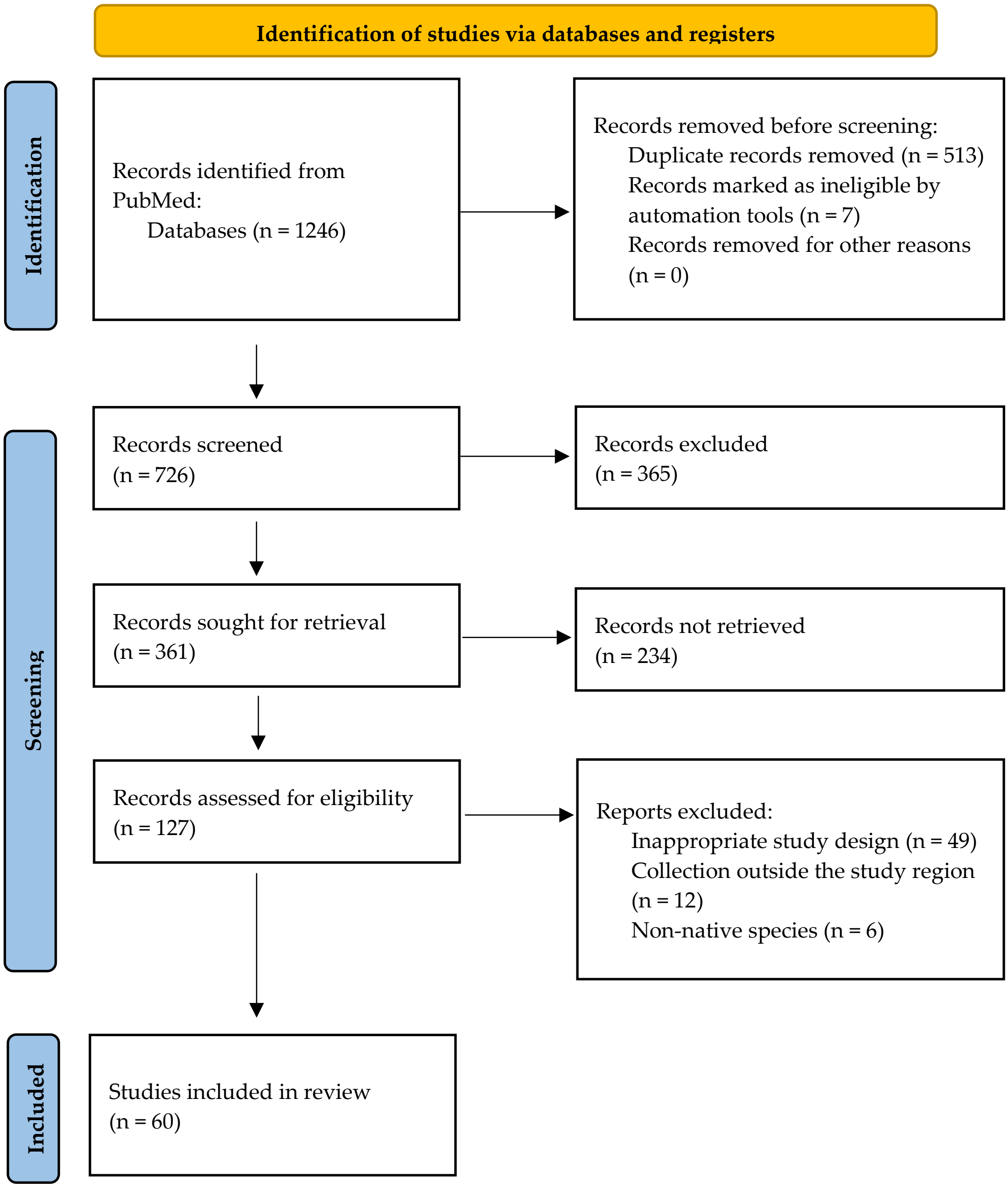

Figure S1. PRISMA Flow Diagram of Study Selection for a Review Characterizing the Bat Virome of Vietnam.
